# Diagnostic Accuracy of Ultrasonography in Revision Thyroid Surgery: Can It Predict Disease Outcomes?

**DOI:** 10.1007/s13193-024-01955-5

**Published:** 2024-06-03

**Authors:** Sasi Krishna Kavutarapu, Suman Kumar Ankathi, Shivakumar Thiagarajan, Anuja Deshmukh, Deepa Nair, Prathamesh Srinivas Pai, Devendra Arvind Chaukar, Gouri Pantvaidya

**Affiliations:** 1 Dept of Head and Neck Surgery, Tata Memorial Hospital, Homi Bhabha National Institute, 12, Thfloorfloor, Homi Bhabha Block, Parel, Mumbai India; 2 Dept of Radiodiagnosis, Tata Memorial Hospital, Homi Bhabha National Institute, Mumbai, India

**Keywords:** Ultrasound, Revision thyroid surgery, Sensitivity, Specificity, Survival

## Abstract

Revision thyroid surgery for residual/recurrent disease is known to have higher complication rates because of parathyroid injury and recurrent laryngeal nerve (RLN) damage. The aim of this study is to evaluate the accuracy of USG in predicting recurrent disease and disease outcomes in patients undergoing reoperation for recurrent/residual thyroid cancer. We performed a retrospective analysis of all thyroid reoperations from 2015 to 2017. Preoperative USG findings were categorized as per prespecified disease stations in the neck and compared with histopathology to calculate sensitivity, specificity, positive predictive value, and negative predictive value of USG. Survival analysis was performed using Kaplan–Meier curves. Two hundred fifty patients were included in the analysis. In a reoperative setting, USG had an overall sensitivity, specificity, accuracy, positive predictive value, and negative predictive value of 89%, 77%, 89%, 94%, and 60%, respectively. We found a significantly lower disease-free survival in patients who had radiologically detected recurrent disease as compared to disease detected only on histopathology. USG has a reasonable accuracy in determining status of lesions in patients undergoing revision thyroid surgeries.

## Introduction

The thyroid gland is one of the most common sites for endocrine malignancy and is the 9th most common cancer [1]. Over the last 25 years, the incidence of thyroid cancers has increased dramatically which has been attributed to improved detection rates [2]. Two-thirds of thyroid cancers are diagnosed with disease confined to the thyroid gland, and such localized thyroid cancers have a 5-year survival rate of almost 100%. Though survivals are high in thyroid cancers, locoregional recurrences are of major concern occurring in 9–30% of patients [3]. The treatment of choice for most locoregional recurrences is surgery followed by radioactive iodine (RAI) therapy if indicated. Revision thyroid surgery for recurrences is known to have higher complication rates, especially with regard to development of hypoparathyroidism because of parathyroid injury and recurrent laryngeal nerve (RLN) damage [4, 5].

Ultrasonography (USG) is the first-line investigation in the workup of a patient with thyroid cancers, both in the treatment naïve and in patients with suspected recurrence/residual disease [6]. However, most of the data regarding the accuracy of ultrasound in thyroid cancers is on treatment naïve patients with a paucity of literature on its accuracy in the recurrent and residual cancer setting. Our primary aim was to evaluate the accuracy of USG in patients with suspected recurrence or residual disease after initial surgery. Our secondary aims were to determine the accuracy of USG at various substations in the neck and disease outcomes in patients undergoing revision surgery for recurrent/residual thyroid cancer.

## Materials and Methods

After obtaining Institutional Review Board approval, a retrospective analysis of all thyroid cancer patients undergoing revision surgery from 2015 to 2017 was performed. Cases were identified from a prospectively maintained institutional surgical database. Patients undergoing resurgery for suspected recurrence in the residual thyroid/thyroid bed/central or lateral nodal compartments, patients undergoing completion surgery for previously operated hemithyroidectomies, and incomplete surgeries in high-risk patients were included. Patients who had undergone surgery for benign thyroid diseases, patients with incomplete operative details, nonavailability of preoperative USG, and those with incomplete preoperative USG findings were excluded from the analysis. Previous operative information, findings of neck sonography done before reoperation, operative procedure, and histopathological findings were reviewed from the prospective database and from hospital electronic medical records.

### Imaging

USG was performed using high-frequency linear array transducers of 7-13 MHz (GE LOGIQ™ E9). At our institution, USGs are performed and interpreted by speciality trained head and neck radiologists. These reports were evaluated and USGs of patients with indeterminate findings were rereviewed by SA^#^.

### Image Interpretation

USG findings were classified into four subsections: thyroid bed, residual lobe, central compartment nodes, and lateral compartment nodes.

*Thyroid bed* was defined as an inverted triangular area with hyperechoic fibrofatty tissue with boundaries as follows: lateral boundary by the carotid arteries, medially by the proximal trachea, ventrally by the strap muscles, craniocaudally from the lower half of the thyroid cartilage to the thoracic inlet.

*Residual lobe*: The remaining thyroid lobe in patients who had undergone prior hemithyroidectomy/isthmectomy.

*Lateral compartment* nodes: Neck nodes were divided into level II to level V according to the imaging based nodal classification [7].

*Central compartment* was defined as the area between the medial borders of both common carotid arteries, bounded superiorly by the inferior border of hyoid done and inferiorly by the superior border of the manubrium. Platysma, trachea, and prevertebral space were the anterior, posteromedial, and posterolateral limits.

Lesions in the thyroid bed with sonographic features like hypoechogenicity, marginal irregularity or spiculation, microcalcification, and a taller-than-wide shape were classified as suspicious in nature. Lateral and central compartment nodes which are well-defined, coffee bean-shaped, and homogeneous with preserved echogenic fatty hilum were classified as reactive lymphoid hyperplasia. Lateral and central compartment nodes with sonographic features like round shape, absence of an echogenic hilum, microcalcifications, hyperechogenicity, cystic change, and peripheral flow on color doppler were classified as metastatic nodes.

USG reports for patients included in the study were reviewed, and all findings were classified as suspicious, benign, or indeterminate. In the case of USG reports showing indeterminate findings, the images were reviewed by SA. Individuals with unresolvable USG findings were documented as indeterminate. Indeterminate findings were excluded from the accuracy analysis.

As per the American Association of Clinical Endocrinologists (AACE) guidelines, postoperatively, patients with corrected serum calcium (Ca^+2^) levels of < 8.6 mg/dl were considered as having temporary hypoparathyroidism [8]. Individuals with low serum Ca^+2^ levels for a duration > 6 months after surgery or those requiring Ca^+2^ supplementation to maintain optimal serum Ca^+2^ levels beyond 6 months were considered as permanently hypoparathyroid.

Follow-up was calculated from the date of reoperation to the last date of follow-up.

### Statistical Analysis

Sensitivity, specificity, positive predictive value, and negative predictive value were calculated using histopathology as the gold standard for the four subsections within the neck. Survival analysis was performed using the Kaplan–Meier curves. SPSS (Statistical Package for the Social Sciences) version 23 was used for data collection and analysis.

## Results

### Patient Characteristics

**Fig. 1:**
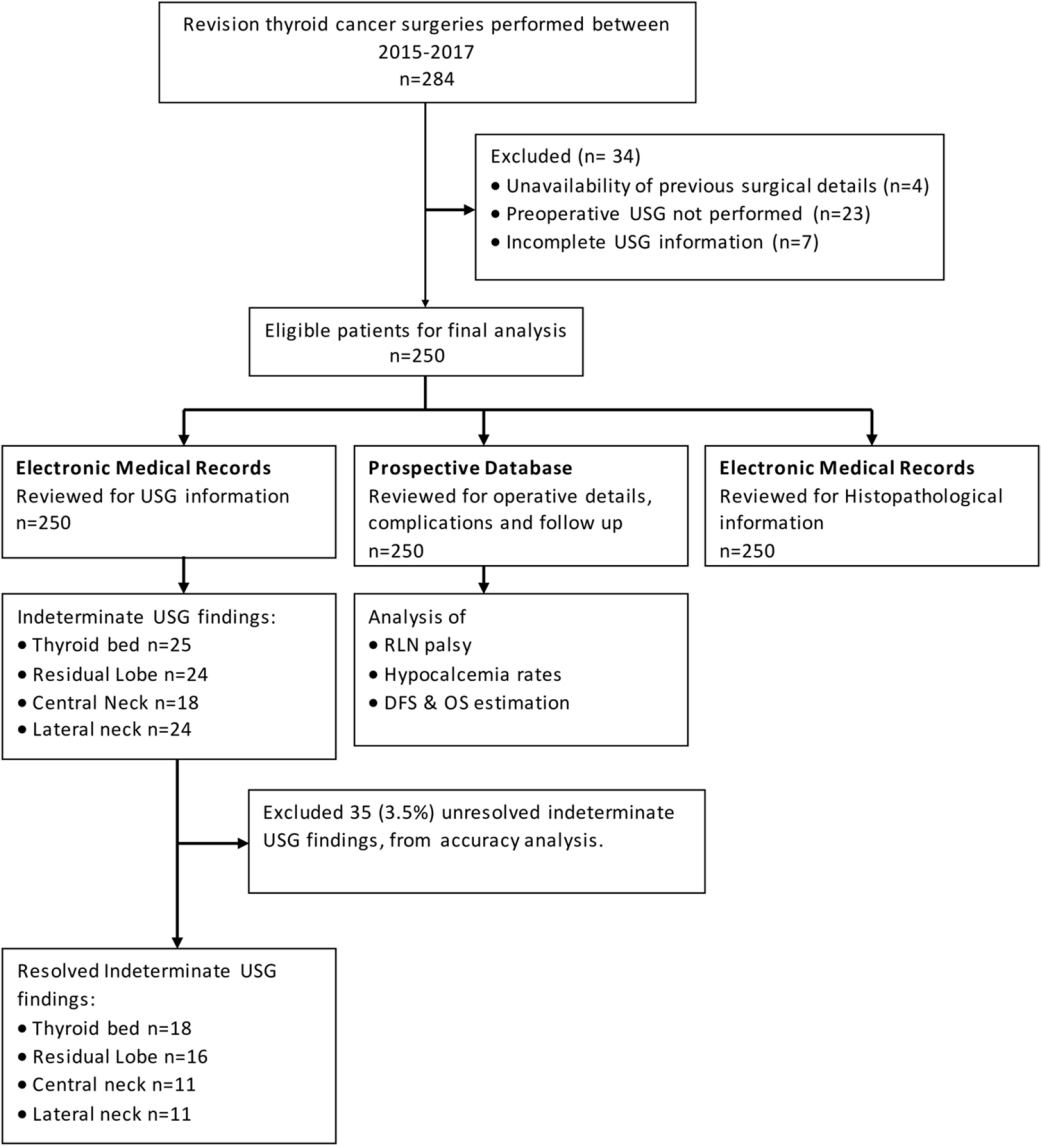
Study flowchart

During the study period, 284 patients underwent reoperation, and 250 were found eligible. The reasons for the exclusion of patients are shown in Fig. 1. Demographic details of the patients included in the analysis are shown in Table 1. There were 157 (62.8%) females and 93 (37.2%) males, with a median age of 40 years. Among the patients presenting to our institution, 198 (71%) were operated at peripheral centers prior to referral to our center.

**Table 1:** Demographic characteristics of 250 patients who had undergone thyroid reoperation

Characteristics	No. of patients (%)
Age	
Median	40 years
Range	15–77 years
Gender	
Male	93 (37.2)
Female	157 (62.8)
Presenting findings	
For completion thyroid lobectomy	113 (45.2)
Recurrence detected on imaging	85 (34)
Lateral neck nodes	35 (14)
Distant metastasis	7 (2.8)
Raised serum thyroglobulin levels	5 (2)
Recurrent thyroid nodule	4 (1.6)
Raised serum calcitonin levels	1 (0.4)
Details of previous surgery	
Hemithyroidectomy	104 (41.6)
Total thyroidectomy + neck dissection	69 (27.6)
Total thyroidectomy	42 (16.8)
Subtotal thyroidectomy	16 (6.4)
Nodule excision Isthmectomy	8 (3.2)
Hemithyroidectomy + neck dissection	6 (2.4)
	5 (2)
No. of previous surgeries	
1	211 (84.4)
2	37 (14.8)
≥ 3	2 (0.8)
Histology	
Papillary thyroid carcinoma	199 (79.6)
Medullary thyroid carcinoma	31 (12.4)
Follicular carcinoma	11 (4.4)
Poorly differentiated carcinoma	7 (2.8)
Hurtle cell carcinoma	2 (0.8)
TNM staging	
T	
T0	161 (64.4)
T1	48 (19.2)
T2	6 (2.4)
T3	24 (9.6)
T4	11 (4.4)
N	
N0	
N1a	99 (39.6)
N1b	32 (12.8)
M	119 (47.6)
M0	234 (93.6)
M1	16 (6.4)
Details of adjuvant treatment prior to thyroid reoperation	
No adjuvant treatment	187 (74.8)
RAI	54 (21.6)
EBRT	7 (2.8)
RAI + EBRT	2 (0.8)

*RAI* radioactive iodine, *EBRT* external beam radiotherapy, *FVPTC* follicular variant of papillary thyroid carcinoma

### Presenting Findings

The most common indication for thyroid reoperation was high-risk disease detected on histopathology after previous surgery (45%), requiring a completion thyroidectomy, to facilitate RAI therapy. Thirty-four percent of patients underwent reoperation for recurrent disease detected through imaging. Patients presenting with clinically evident lateral neck nodes and distant metastases as their presenting symptoms constituted 14% and 2.8%, respectively. The most common histology was papillary thyroid cancer (79.6%).

### Previous Treatment

Prior to reoperation, 139 (55.2%) of patients had undergone some form of a partial thyroidectomy. Two hundred and eleven (84.4%) patients had undergone only one prior surgery and 37 patients (14.8%) had undergone two surgeries previously. Central compartment nodes were dissected in 25% of patients in the previous surgery. Fifty-six (22.4%) patients had received ablative radioactive ^131^Iodine.

### Reoperations

Of the 250 patients undergoing re surgery, 182 patients underwent reoperative thyroid procedures, 191 selective neck dissections, and 43 super selective node excisions of involved stations.

### Preoperative Sonography

The preoperative USG findings for the four subsections and their correlation with histopathology are shown in Table 2. After an initial review of USG reports, there were 91 indeterminate findings (thyroid bed, 25; residual lobe, 24; central neck, 18; lateral neck, 24). USG images of these indeterminate findings were reviewed and 61.5% could be resolved. There were 35 unresolvable USG findings which were labelled as indeterminate (thyroid bed, 6; residual lobe, 6; central neck, 8; lateral neck, 15) accounting for 3.5% of overall findings and were excluded from sensitivity analysis. Overall, 196 (78.4%) patients had proven malignancy on histopathology. Of the 54 patients who underwent revision surgery with no tumor on histopathology, 39 were completion lobectomies to facilitate RAI therapy in high-risk tumors. The sensitivity, specificity, positive predictive value (PPV), and negative predictive value (NPV) of USG for detecting persistent/recurrent disease in the thyroid bed, residual thyroid lobe, central and lateral compartment nodes are shown in Table 3. USG had the highest sensitivity for detecting disease in the lateral neck, followed by the thyroid bed. The PPV and NPV of USG to detect malignant central compartment nodes in a reoperative setting was 71 and 75%, respectively.

**Table 2:** Preoperative sonographic findings and histopathology correlation

Site and radiologic finding	No. of patients	No. of patients with positive histopathology (%)
Thyroid bed		
Suspicious	51	34 (66.7)
Negative	192	12 (6.3)
Indeterminate	7	2 (28.6)
Residual lobe		
Suspicious	27	23 (85.2)
Negative	215	31 (14.4)
Indeterminate	8	5 (62.5)
Central compartment nodes		
Suspicious	85	60 (70.6)
Negative	158	40 (25.3)
Indeterminate	7	4 (57.1)
Lateral compartment nodes		
Suspicious	109	99 (90.8)
Negative	128	16 (12.5)
Indeterminate	13	7 (53.8)

**Table 3:** Accuracy of USG in evaluation of thyroid recurrences or residual disease at different sites of neck

Characteristic	Site of neck			
	Thyroid bed	Residual thyroid lobe	Central compartment	Lateral neck
Sensitivity (%)	74	43	60	86
Specificity (%)	91	98	83	91
Positive predictive value (%)	67	85	71	90
Negative predictive value (%)	94	86	75	88
Accuracy (%)	88	86	73	89

### Complications

#### RLN Paralysis

In our cohort of 250 patients and 267 nerves at risk (NAR), the temporary RLN palsy rate was 11.2%. Six RLN (2.2%) were sacrificed during surgery because of involvement by disease. Of the 30 RLN which were paralyzed after surgery, 16 nerves (53.3%) regained function at the end of 1 year. Fourteen patients had no documentation of vocal cord function on follow-up, in the hospital records.

#### Hypoparathyroidism

Postoperative Ca^+2^ levels were available in 189 of the 219 patients at risk of hypocalcemia. Hypoparathyroidism was present in 24 (12.7%) patients before reoperation and were on calcium supplements before reoperation. Temporary hypoparathyroidism was seen in 34.9% after reoperation and in 24% after excluding patients who were preoperatively hypoparathyroid.

### Follow-up and Disease Outcomes

Among the 250 patients in our cohort, 207 (82.8%) patients received some form of adjuvant treatment. RAI, RAI with EBRT, EBRT alone, and other therapy like PRRT/TKI therapy were administered in the adjuvant setting in 72%, 17.2%, 2.4%, 7.3%, and 1.2%, respectively. The median follow-up for the cohort was 32 months (range 1–58 months). There was one death in the study cohort, but this was not related to thyroid cancer.

**Fig. 2:**
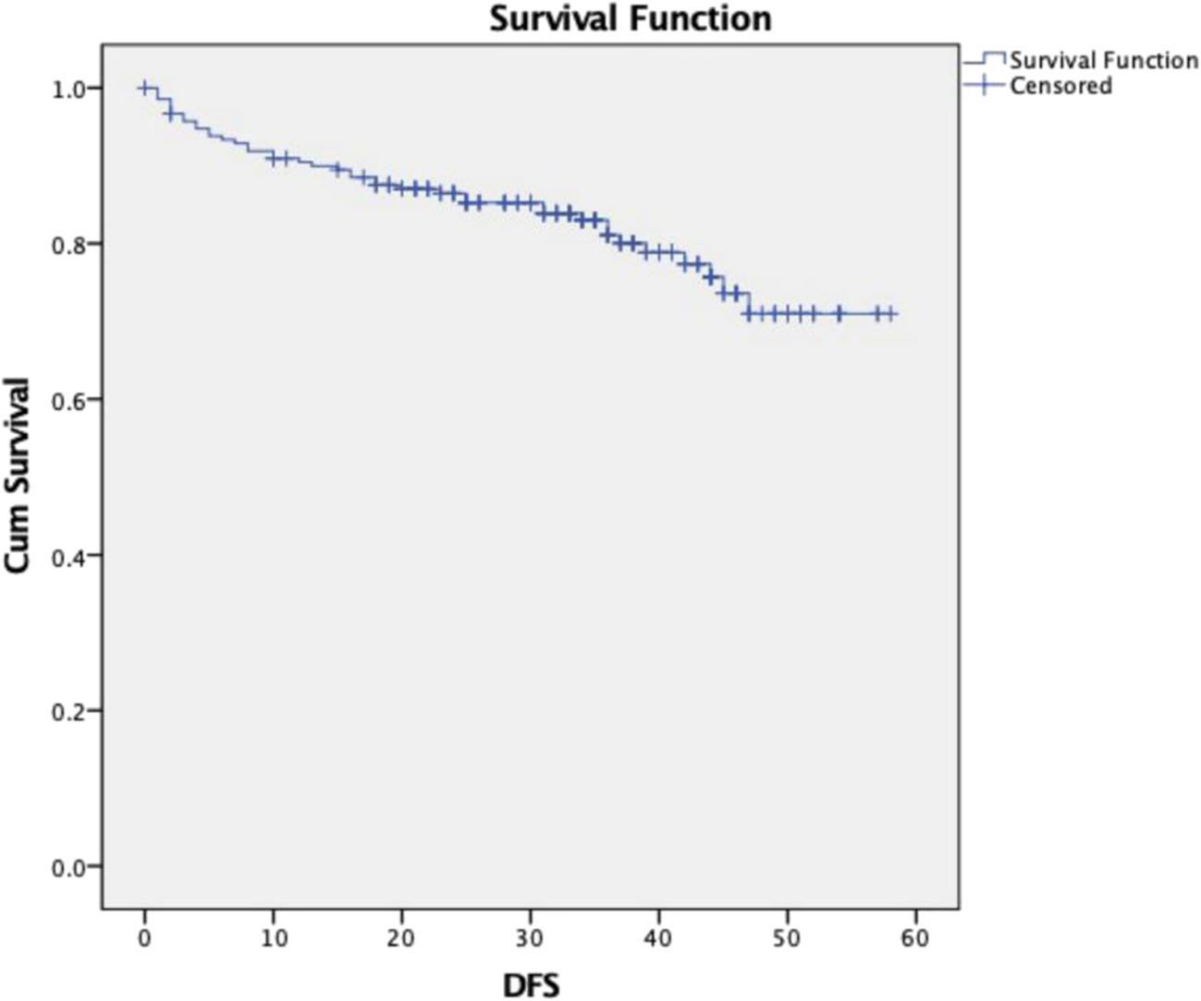
Disease-free survival (DFS)

**Fig. 3:**
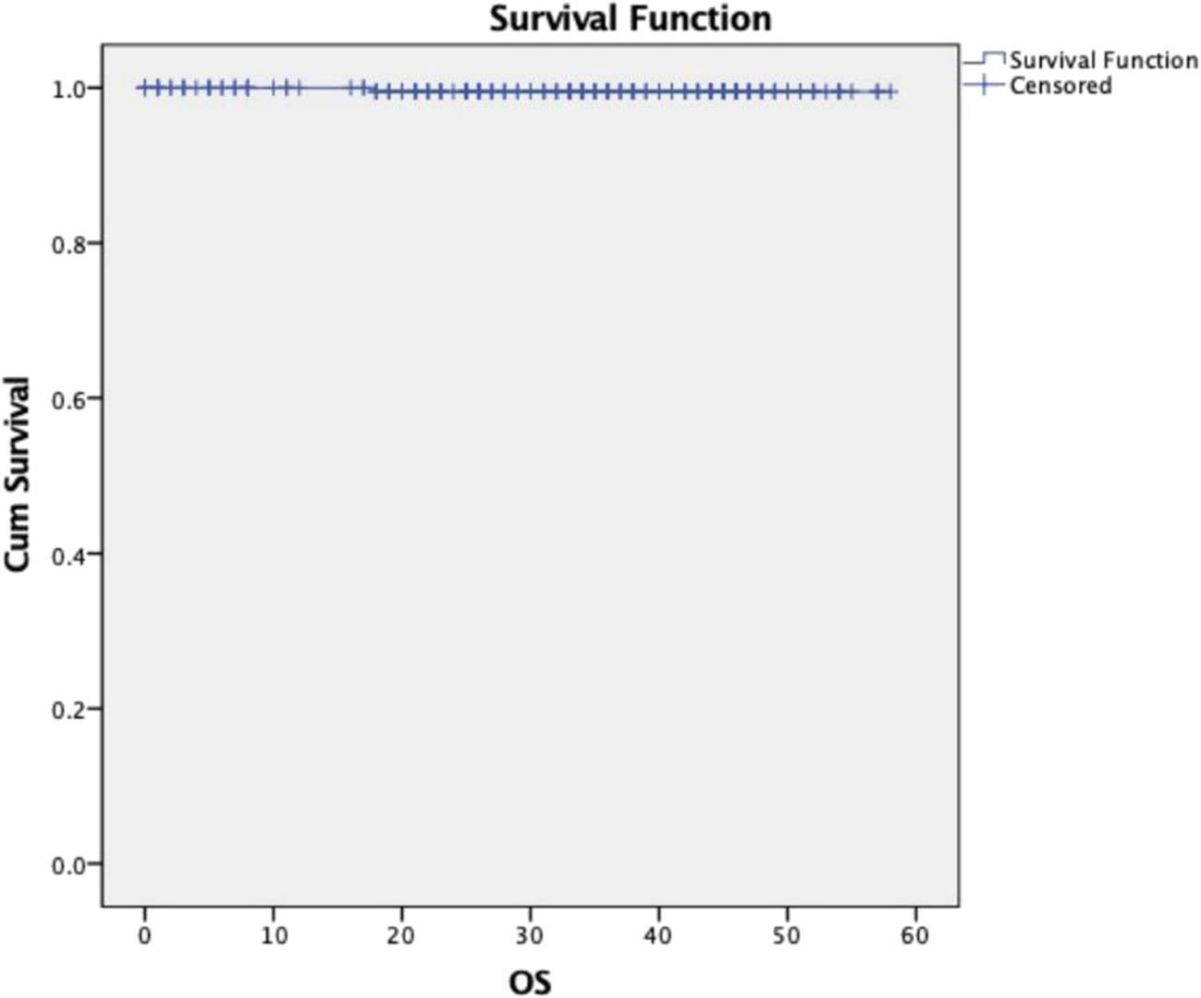
Overall survival (OS)

Recurrences were seen in 41 patients (16.4%) in the cohort. Patterns of recurrence after revision thyroid surgery and their management are shown in Table 4. The most common site of recurrence was the regional lymph nodes, followed by distant metastases. Of the recurrences, 63.4% occurred within 18 months following revision surgery. Five-year disease-free and overall survival was 71% and 99.5%, respectively (Figs. 2 and 3).

**Table 4:** Pattern of recurrences and treatment

Pattern of recurrence	No. of patients	Treatment of recurrences
Local (thyroid bed)	3	Surgery - 2 Observation - 1
Regional (central and lateral neck)	23	Surgery - 13 Observation - 3 RAI - 2 EBRT - 1 TKI - 1 Lost to follow-up - 2 PRRT - 1
Distant	15	Surgery - 1 Observation - 2 RAI - 4 Pall RT - 2 PRRT - 1 TKI - 5

*RAI* radioactive Iodine, *EBRT* external beam radiotherapy, *TKI* tyrosine kinase inhibitors, *PRRT* peptide receptor radionuclide therapy, *Pall RT* palliative radiotherapy

### Comparison of Disease-Free Survival Within Subgroups

**Fig. 4:**
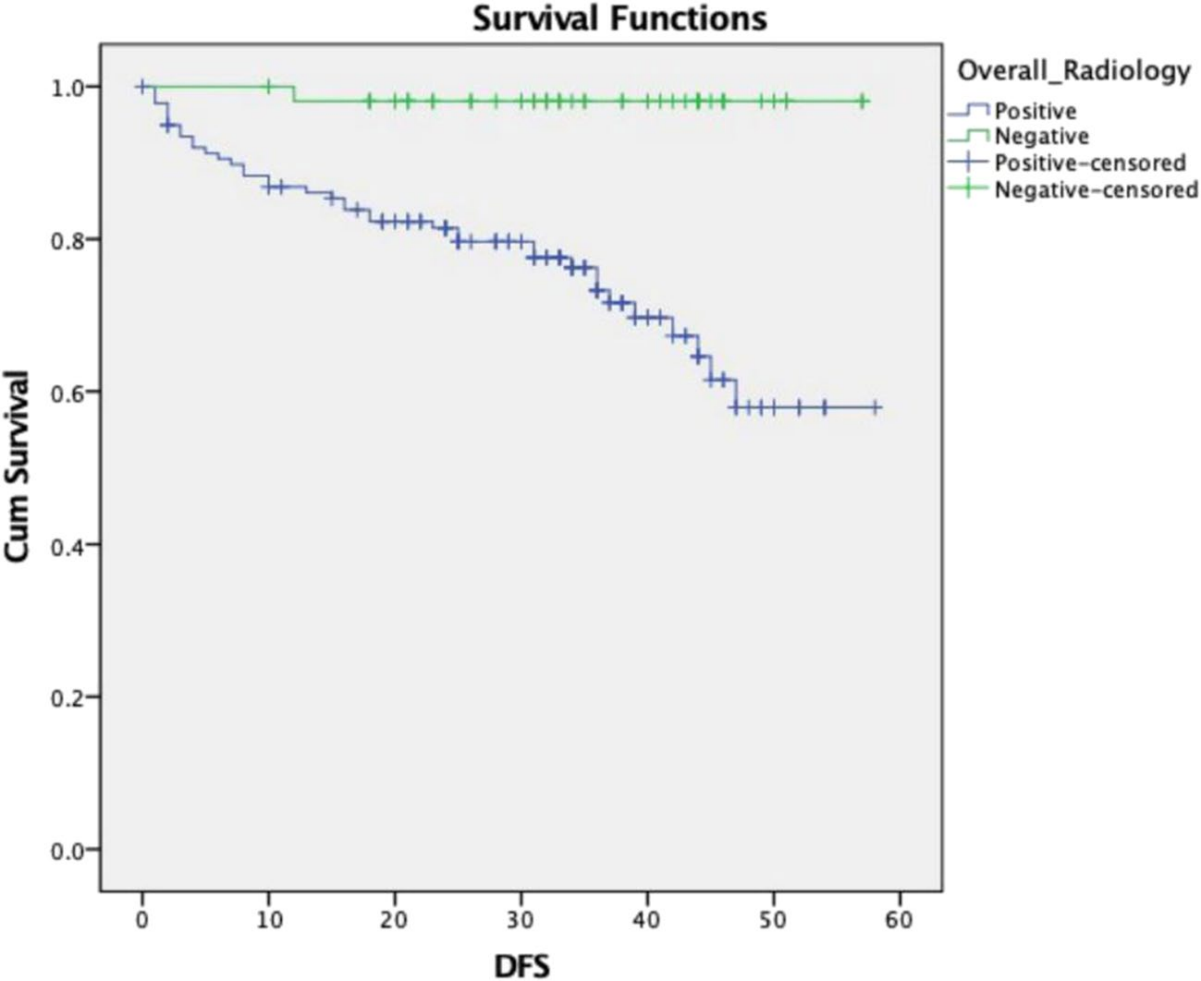
Disease-free survival (DFS) according to radiological subgroups

Patients with the radiological positive disease had a worse 5-year DFS (58%) as compared to radiologically negative individuals (98%). This difference was statistically significant (*p* value: 0.0001). Figure 4 shows the Kaplan–Meier graph demonstrating the survival difference.

## Discussion

USG is a noninvasive tool that has been the first-line investigation in the workup of thyroid cancers. There is abundant literature pertaining to the accuracy of USG in detecting disease prior to initial operation [9, 10]. However, literature on the accuracy of USG in revision thyroid surgeries is sparse. Prior surgery leaves a nonvirgin field, distorts normal anatomy, and makes it challenging to identify vital structures like the RLN and parathyroid glands. The nerves can lie in a less predictable location after thyroidectomy, or the nerve and the glands may be encased within fibrotic tissue, making it difficult to distinguish these from a tumor [11]. Reoperations in the thyroid bed/central compartment carry a risk of damage to RLN and parathyroid glands. Revision surgery for thyroid cancer can be morbid and significantly affect the quality of life in thyroid cancers, where the survival rates are above 90% in most differentiated cancers.

A review of the literature found 6 publications (Table 5) reporting the accuracy of USG in detecting residual/recurrent thyroid cancers [12–17]. Studies by Stulak et al. and Shin et al. had evaluated the performance of USG in detecting nodal metastases and surgical bed recurrences, respectively. However, most of these studies have reported cumulative sensitivity and specificity of USG in all subsites of the neck. As anatomy in revision cases may be distorted, both for the radiologist and the surgeon and it is vital to surgically address only areas where recurrence/persistent disease is suspected, we calculated the sensitivity, specificity, positive predictive value, and negative predictive value at individual subsites of the neck, i.e., the thyroid bed, residual thyroid lobe, central compartment, and lateral neck. The overall sensitivity and specificity of USG in our study are in concordance with that reported by Onkendi et al. and Stulak et al. [13, 16]. Results of our study show that USG has high specificity (83–98%) and a variable sensitivity (43–86%) at various levels of the neck. In a reoperative setting, USG has a sensitivity of 74% and 60% respectively in the thyroid bed and the central compartment. These modest detection rates may warrant the addition of computed tomography (CT) or a USG-guided FNAC in revision cases to avoid unnecessary reoperations. However, the literature for the same is not adequate, and a study by Hong et al. recommends combining CT and USG to increase sensitivity in detecting cervical recurrence in thyroid cancers [18]. A meta-analysis by Zhao et al. reported a sensitivity of 33%, 70%, specificity of 93%, 84% by USG, in detecting central and lateral neck nodes respectively among previously untreated patients with papillary thyroid cancer [19]. Similarly, in a recent systematic review and meta-analysis looking at a comparison of USG and CT in per primum cases, Alabousi et al. reported a sensitivity of 28%, 73%, specificity of 95%, 89% for USG in detecting central and lateral neck nodes respectively. Overall, there was no significant difference between CT and USG. However, CT showed better results in detecting central neck nodes and this study was performed only on treatment naïve population [20]. Our study had shown USG to be more sensitive in detecting central and lateral neck nodal recurrences with a sensitivity of 60% and 86% respectively and a specificity of 83% and 91% respectively, akin to that reported in previously untreated individuals. Also, our study found a significantly different DFS in patients who had radiologically detected recurrent disease as compared to patients with negative USG (58% vs. 98%). The poor DFS in patients with suspicious findings on USG may also be attributed to the inherent aggressive biology of the disease. However, this finding further underlines the need for accurate radiologic diagnosis in patients suspected to have recurrent/residual disease. The OS and DFS in our study were 99.5 and 71%, respectively with a median follow-up of 32 months. The median follow-up in our study was short for differentiated thyroid cancers; however, 51% of recurrences in our cohort occurred within 18 months of surgery. A study by Palme et al. evaluated 574 patients with a median follow-up of 7 years. They reported an actuarial disease-specific survival at 20 years of 100%, 94%, and 60% in patients with no recurrence, one recurrence, and multiple recurrences, respectively [21]. Literature on thyroid reoperations for residual/recurrent disease report a RLN palsy rate of 0–25% and a hypocalcemia rate of 0–14.6% [22–25]. Publications with a minimum sample size of 100 quote a RLN palsy rate of 0–3% and a hypocalcemia rate of 10% [16, 26]. Gimm et al. and Pantvaidya et al. had reported RLN palsy > 10% in their cohort of revision cases [25, 27]. Patients having RLN palsy after revision surgery have also been shown to have a longer recovery time as compared to per primum surgery [24].

**Table 5:** Literature review of USG accuracy in preoperative evaluation of reoperations for thyroid cancers

Author	Type	Year	*N*	Sensitivity	Specificity	Positive predictive value	Negative predictive value	Accuracy
Frasoldati et al. [12]	Retrospective	2003	51	92%	90%	-	-	90%
De Rosario et al. [13]	Prospective	2004	81	96%	97%	-	-	93%
Stulak et al. [14]	Retrospective	2006	219	90%	79%	94%	-	88%
Shin et al. [15]	Retrospective	2007	58	79%	97%	94%	90%	-
Lepoutre-Lussey et al. [16]	Retrospective	2014	138	83%	87%	96%	61%	-
Onkendi et al. [17]	Retrospective	2014	399	93%	80%	97%	-	92%
Our study	Retrospective	2022	250	89%	77%	94%	60%	89%

*N* number of individuals

Our study has some limitations inherent to the retrospective nature of the study. The subset of patients who underwent USG but did not undergo revision surgery were not included in this study. This may lead to inherent bias in the calculation of the accuracy of USG. Also, a large majority of patients (84%) in our study were only operated once previously. Hence, our results may not apply to a cohort of patients who have undergone multiple surgeries with further distortion of anatomy. Indeterminate findings were not included in the accuracy analysis and their significance remains unanswered. However, these indeterminate findings constituted a small proportion (3.5%) of results in the study. In a publication by Shin et al., most recurrent tumors had nonspecific findings like well-defined, oval, hypoechoic nodules on USG and such lesions could have been missed in our study [15]. In a surgical bed after thyroidectomy, normal structures like residual thyroid, parathyroid glands, suture granulomas, and lymph nodes can be misdiagnosed as recurrent tumor. Nonrecurrent lesions like suture granuloma, strap muscle, postoperative fibrosis, and degenerated cysts can present as nodules with punctate echogenic foci on USG mimicking recurrent tumors which show microcalcifications [28]. A USG-guided FNAC differentiates such nonrecurrent lesions from recurrent tumors. This additional benefit of USG-guided FNAC has not been evaluated in our study and USG with FNAC may further improve the accuracy of USG in patients undergoing revision thyroid surgery.

## Conclusion

USG has a reasonable accuracy in determining the status of lesions in patients undergoing revision surgeries for thyroid cancers. However, there may be a case in point to add another modality like a CT or USG-guided FNAC to improve the determination of disease in the reoperated neck. This may help decrease unnecessary revision surgery and its attendant complications in patients with long survival and good disease outcomes.

## Data Availability

The data that support the findings of this study are not openly available due to reasons of sensitivity and are available from the corresponding author upon reasonable request.
